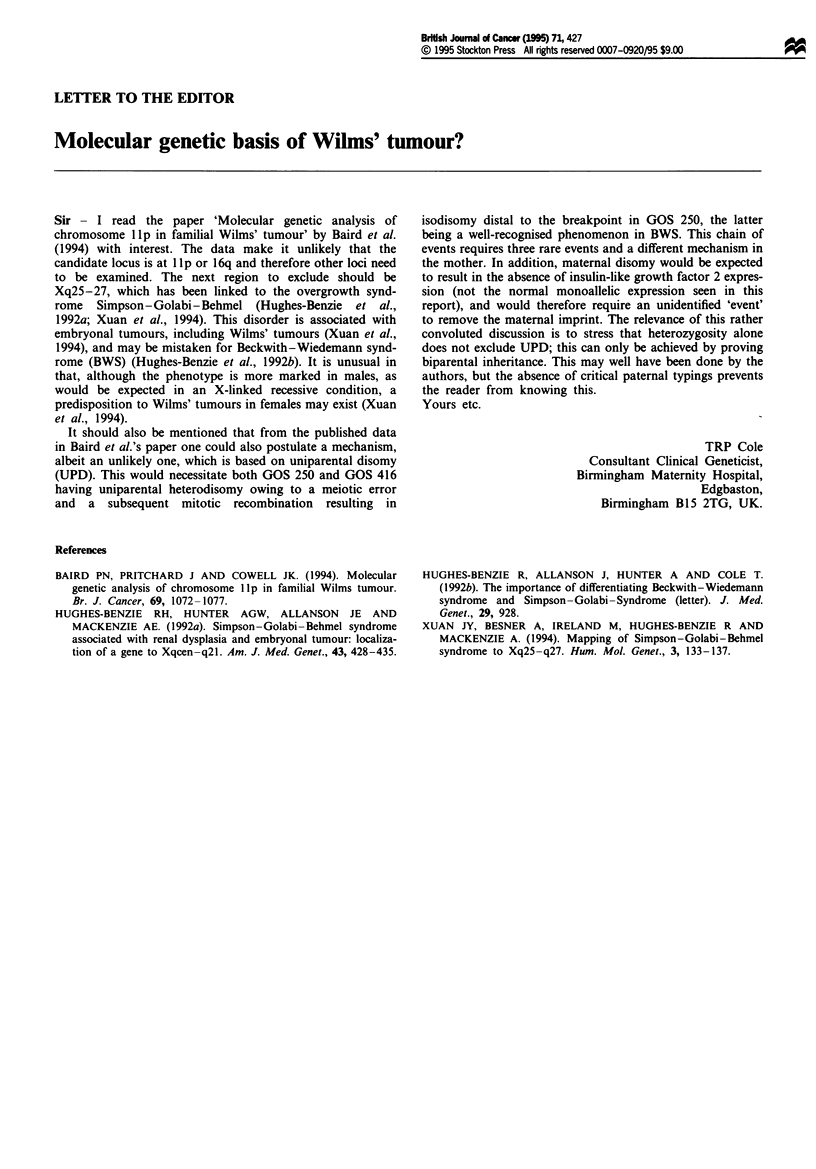# Molecular genetic basis of Wilms' tumour?

**DOI:** 10.1038/bjc.1995.87

**Published:** 1995-02

**Authors:** T. R. Cole


British Journal of Cancer (1995) 71, 427
© 1995 Stockton Press All rights reserved 0007-0920/95 $9.00

LETTER TO THE EDITOR

### Molecular genetic basis of Wilms' tumour?

Sir – I read the paper 'Molecular genetic analysis of chromosome 11p in familial Wilms' tumour' by Baird *et al.* (1994) with interest. The data make it unlikely that the candidate locus is at 11p or 16q and therefore other loci need to be examined. The next region to exclude should be Xq25–27, which has been linked to the overgrowth syndrome Simpson–Golabi–Behmel (Hughes-Benzie *et al.*, 1992*a*; Xuan *et al.*, 1994). This disorder is associated with embryonal tumours, including Wilms' tumours (Xuan *et al.*, 1994), and may be mistaken for Beckwith–Wiedemann syndrome (BWS) (Hughes-Benzie *et al.*, 1992*b*). It is unusual in that, although the phenotype is more marked in males, as would be expected in an X-linked recessive condition, a predisposition to Wilms' tumours in females may exist (Xuan *et al.*, 1994).

It should also be mentioned that from the published data in Baird *et al.*'s paper one could also postulate a mechanism, albeit an unlikely one, which is based on uniparental disomy (UPD). This would necessitate both GOS 250 and GOS 416 having uniparental heterodisomy owing to a meiotic error and a subsequent mitotic recombination resulting in isodisomy distal to the breakpoint in GOS 250, the latter being a well-recognised phenomenon in BWS. This chain of events requires three rare events and a different mechanism in the mother. In addition, maternal disomy would be expected to result in the absence of insulin-like growth factor 2 expression (not the normal monoallelic expression seen in this report), and would therefore require an unidentified 'event' to remove the maternal imprint. The relevance of this rather convoluted discussion is to stress that heterozygosity alone does not exclude UPD; this can only be achieved by proving biparental inheritance. This may well have been done by the authors, but the absence of critical paternal typings prevents the reader from knowing this.

Yours etc.

TRP Cole
Consultant Clinical Geneticist,
Birmingham Maternity Hospital,
Edgbaston,
Birmingham B15 2TG, UK.

### References

BAIRD PN, PRITCHARD J AND COWELL JK. (1994). Molecular genetic analysis of chromosome 11p in familial Wilms tumour. *Br. J. Cancer*, **69**, 1072–1077.

HUGHES-BENZIE RH, HUNTER AGW, ALLANSON JE AND MACKENZIE AE. (1992*a*). Simpson–Golabi–Behmel syndrome associated with renal dysplasia and embryonal tumour: localization of a gene to Xqcen–q21. *Am. J. Med. Genet.*, **43**, 428–435.

HUGHES-BENZIE R, ALLANSON J, HUNTER A AND COLE T. (1992*b*). The importance of differentiating Beckwith–Wiedemann syndrome and Simpson–Golabi–Syndrome (letter). *J. Med. Genet.*, **29**, 928.

XUAN JY, BESNER A, IRELAND M, HUGHES-BENZIE R AND MACKENZIE A. (1994). Mapping of Simpson–Golabi–Behmel syndrome to Xq25–q27. *Hum. Mol. Genet.*, **3**, 133–137.